# Supplementary material for: Determinants of rice production and market supply: A study of Bench Sheko zone in Ethiopia
Source: PLoS One. 2024 Sep 6;19(9):e0302115. doi: 10.1371/journal.pone.0302115 (PMC11379224; doi:10.1371/journal.pone.0302115)
Supplement: Attachment — Submitted filename: PONE-D-24-11303.pdf [file pone.0302115.s002.pdf]

# PLOS ONE

# Factors Affecting Rice Production and Market Supply: the case of Guraferda district, Bench Maji zone in Southern Ethiopia

--Manuscript Draft--

| | |
|---|---|
| **Manuscript Number:** | PONE-D-24-11303 |
| **Article Type:** | Research Article |
| **Full Title:** | Factors Affecting Rice Production and Market Supply: the case of Guraferda district, Bench Maji zone in Southern Ethiopia |
| **Short Title:** | Factors Affecting Rice Production and Market Supply |
| **Corresponding Author:** | Ejigu Mulatu<br>Policy Studies Institute<br>Addis Ababa, ETHIOPIA |
| **Keywords:** | Linear regression model, market supply, rice production constraints, rice technologies |
| **Abstract:** | In Ethiopia rice crop is considered as a strategic food security crop which is expected to contribute in ensuring food security in the country. Guraferda district is one of a major rice growing area in Bench Maji zone under Southern National Nationalities and Peoples Regional State at large. The study was conducted with specific objectives to investigate factor affecting smallholder farmers' market supply of rice and identify constraints related with rice production in the study area. Two-stage sampling technique was employed to select 119 representative rice producer households. Both descriptive statistics and econometric model were used to analyze the data. Multiple linear regression model used to analyze factors affecting rice market supply. Farm size owned, credit use, Annual income, number of oxen owned and quantity of rice produced were found to be significantly affecting market supply of rice in a district. Major constraints related with rice production on the district were lack of proper weed management practices, improved seed, proper method and time of fertilizer application, weak institutional support, disease and post-harvest handling problems were also important. The research findings suggest that, attention should be given on rice production constraints through generation and wide demonstration of demand-driven rice production and post- harvest handling technologies for increased production and productivity to have better market supply of rice to the market and benefit smallholder farmers |
| **Order of Authors:** | Ejigu Mulatu |
| **Opposed Reviewers:** | |
| **Additional Information:** | |
| **Question** | **Response** |
| **Financial Disclosure**<br><br>Enter a financial disclosure statement that describes the sources of funding for the work included in this submission. Review the submission guidelines for detailed requirements. View published research articles from *PLOS ONE* for specific examples.<br><br>This statement is required for submission and **will appear in the published article** if the submission is accepted. Please make sure it is accurate. | The author(s) received no specific funding for this work. |

Powered by Editorial Manager® and ProduXion Manager® from Aries Systems Corporation

| | |
|---|---|
| **Funded studies**<br>Enter a statement with the following details:<br>• Initials of the authors who received each award<br>• Grant numbers awarded to each author<br>• The full name of each funder<br>• URL of each funder website<br>• Did the sponsors or funders play any role in the study design, data collection and analysis, decision to publish, or preparation of the manuscript?<br><br>Did you receive funding for this work? | |
| **Competing Interests**<br><br>Use the instructions below to enter a competing interest statement for this submission. On behalf of all authors, disclose any competing interests that could be perceived to bias this work—acknowledging all financial support and any other relevant financial or non-financial competing interests.<br><br>This statement is required for submission and **will appear in the published article** if the submission is accepted. Please make sure it is accurate and that any funding sources listed in your Funding Information later in the submission form are also declared in your Financial Disclosure statement.<br><br>View published research articles from *PLOS ONE* for specific examples. | The author have declared that no competing interests exist. |

*Powered by Editorial Manager® and ProduXion Manager® from Aries Systems Corporation*

| | |
|---|---|
| **NO authors have competing interests**<br><br>Enter: *The authors have declared that no competing interests exist.*<br><br>**Authors with competing interests**<br><br>Enter competing interest details beginning with this statement:<br><br>*I have read the journal's policy and the authors of this manuscript have the following competing interests: [insert competing interests here]*<br><br>* typeset | |
| **Ethics Statement**<br><br>Enter an ethics statement for this submission. This statement is required if the study involved:<br><br>• Human participants<br>• Human specimens or tissue<br>• Vertebrate animals or cephalopods<br>• Vertebrate embryos or tissues<br>• Field research<br><br>Write "N/A" if the submission does not require an ethics statement.<br><br>General guidance is provided below. Consult the submission guidelines for detailed instructions. **Make sure that all information entered here is included in the Methods section of the manuscript.** | Since the financing institution approved the study proposal, the study was based on cross-sectional data collected from rice producer households at a time. Every sample participant gave verbal consent before the interview and not consented participants were not scheduled for interview. No written ethics approval was needed to conduct the survey as the usual working procedure of the country. The consent form asked is shown below.<br><br>Consent Form<br>Introduction:<br>My name is [INTERVIEWER name], and I work for the southern agricultural research institute as a data collector. We have been appointed by the institute to conduct a study on rice production and marketing. We aim to understand factors affecting rice production and market supply to identify major improvement areas. We thank you for your time to participate in this survey. To ensure the accuracy and effectiveness of our study, we have obtained a list of all rice producing farmers from the farmers' association group. We then randomly selected sample producers to participate in the study. You have been selected from this list, and we cordially invite you to participate in the study. Your participation will greatly contribute to our understanding of the problems related with rice production and market supply, so your input is particularly valuable to us.<br>Participation in this interview is voluntary, and you may choose not to take part or to withdraw from the interview at any time without any consequences. Your decision will not affect your relationship with the institute or farmers' association leaders.<br><br>Risks and Benefits:<br>There is no identified risk related with participation in this study. However, some questions may be personal and, and you may feel uncomfortable answering them. If you feel any distress, we advise you to seek support from a friend you trust. There is no any direct benefit provided to you for participating in this interview. However, your participation will contribute to a better understanding of the rice production and marketing related challenges, which may help improve the policy support.<br><br>Confidentiality:<br>All information you provide will be kept strictly confidential. We will not share your name and other identifying information with anyone outside the research team. Your |

| | |
|---|---|
| **Format for specific study types**<br><br>**Human Subject Research (involving human participants and/or tissue)**<br>• Give the name of the institutional review board or ethics committee that approved the study<br>• Include the approval number and/or a statement indicating approval of this research<br>• Indicate the form of consent obtained (written/oral) or the reason that consent was not obtained (e.g. the data were analyzed anonymously)<br><br>**Animal Research (involving vertebrate animals, embryos or tissues)**<br>• Provide the name of the Institutional Animal Care and Use Committee (IACUC) or other relevant ethics board that reviewed the study protocol, and indicate whether they approved this research or granted a formal waiver of ethical approval<br>• Include an approval number if one was obtained<br>• If the study involved *non-human primates*, add *additional details* about animal welfare and steps taken to ameliorate suffering<br>• If anesthesia, euthanasia, or any kind of animal sacrifice is part of the study, include briefly which substances and/or methods were applied<br><br>**Field Research**<br><br>Include the following details if this study involves the collection of plant, animal, or other materials from a natural setting:<br>• Field permit number<br>• Name of the institution or relevant body that granted permission | responses will be assigned a unique code number to maintain your anonymity."<br><br>Contact Information:<br>If you have any questions about this study or your participation in it, please get in touch with the research team at [Ejigu Mulatu at 0910140961]<br><br>Do you think that you would be interested in participation? Would you allow us to keep in touch even after this interview? May I proceed with the interview?<br><br>Yes [CONTINUE WITH INTERVIEW]<br>No [END INTERVIEW]<br><br>If the respondent gives consent the interview continues and if not that was the end of the interview. |
| **Data Availability**<br><br>Authors are required to make all data underlying the findings described fully available, without restriction, and from the time of publication. PLOS allows rare exceptions to address legal and ethical concerns. See the PLOS Data Policy and FAQ for detailed information. | Yes - all data are fully available without restriction |

| | |
|---|---|
| A Data Availability Statement describing where the data can be found is required at submission. Your answers to this question constitute the Data Availability Statement and **will be published in the article**, if accepted.<br><br>**Important:** Stating 'data available on request from the author' is not sufficient. If your data are only available upon request, select 'No' for the first question and explain your exceptional situation in the text box.<br><br>Do the authors confirm that all data underlying the findings described in their manuscript are fully available without restriction? | |
| **Describe where the data may be found in full sentences. If you are copying our sample text, replace any instances of XXX with the appropriate details.**<br><br>• If the data are **held or will be held in a public repository**, include URLs, accession numbers or DOIs. If this information will only be available after acceptance, indicate this by ticking the box below. For example: *All XXX files are available from the XXX database (accession number(s) XXX, XXX.*).<br>• If the data are all contained **within the manuscript and/or Supporting Information files**, enter the following: *All relevant data are within the manuscript and its Supporting Information files.*<br>• If neither of these applies but you are able to provide **details of access elsewhere**, with or without limitations, please do so. For example:<br><br>*Data cannot be shared publicly because of [XXX]. Data are available from the XXX Institutional Data Access / Ethics Committee (contact via XXX) for researchers who meet the criteria for access to confidential data.*<br><br>*The data underlying the results presented in the study are available from (include the name of the third party* | Data will be available upon the request |

| | |
|---|---|
| *and contact information or URL).*<br>• This text is appropriate if the data are owned by a third party and authors do not have permission to share the data.<br><br>* typeset | |
| Additional data availability information: | |

Powered by Editorial Manager® and ProduXion Manager® from Aries Systems Corporation

**Title:** Factors Affecting Rice Production and Market Supply: the case of Guraferda district, Bench Maji zone in Southern Ethiopia

Names of author: Ejigu Mulatu

Name of the corresponding author: Ejigu Mulatu

Type of manuscript: Research Article

I am resubmitting the manuscript revised based on the comment on edits required. The manuscript is original research article prepared on important crop rice. Rice is considered as important food and livelihood security crop in Ethiopia. Previous studies solely focused on biological aspects and measuring efficiency level, showing little about factors affecting production and marketing of rice output. This study would add value by assessing production and marketing-related issues together. The findings of the research would be important source of information for evidence-based policy formulation. I am interested in PLOS ONE Journals as it covers wide scope in publishing scientific articles including social science. Therefore, the manuscript is appropriate for Life science Journal in category of Agricultural Economics.

I confirm that the manuscript, or its contents in some other form, has not been published previously by the author and/or is not under consideration for publication in another journal at the time of submission.

Manuscript

Click here to access/download;Manuscript;Rice paper modified March 23 .docx

# Factors Affecting Rice Production and Market Supply: the case of Guraferda district, Bench Maji zone in Southern Ethiopia

Ejigu Mulatu

Policy Studies Institute, P. O. Box 2479, Addis Ababa, Ethiopia

Tel: +251910140961 Email: ejeta2010@gmail.com

## ABSTRACT

*In Ethiopia rice crop is considered as a strategic food security crop which is expected to contribute to ensuring food security in the country. Guraferda district is one of the major rice growing areas in the Bench Maji zone under Southern National Nationalities and Peoples Regional State at large. The study was conducted with specific objectives to investigate factors affecting smallholder farmers' market supply of rice and identify constraints related to rice production in the study area. Two-stage sampling technique was employed to select 119 representative rice-producer households. Descriptive statistics and appropriate econometric models were used to analyze the collected data. Multiple linear regression model used to analyze factors affecting rice market supply. Farm size owned, credit use, Annual income, number of oxen owned, and quantity of rice produced were found to be significantly affecting the market supply of rice in a district. Major constraints related to rice production in the district were a lack of proper weed management practices, improved seed, proper method and time of fertilizer application, weak institutional support, disease, and post-harvest handling problems were also important. The research findings suggest that attention should be given to rice production constraints through generation and wide demonstration of demand-driven rice production and post-harvest handling technologies for increased production and productivity to have a better market supply of rice to the market and benefit smallholder farmers.*

**Keywords**: Linear regression model, market supply, rice production constraints, rice technologies

## 1. INTRODUCTION

Globally, rice production has risen to 758.8 million tons of paddy rice. In addition to its contribution to consumption, it is a good opportunity for domestic and international markets for economic development mainly in China, India Indonesia, and USA, and other countries [1,2]. Rice is the most significant food crop in the world and it has been considered to assist as a chief food basis for more than 50% of the world's population for the long years [3]. Due to these reasons, it has got great attention from different stakeholders.

Rice was introduced to Ethiopia in the late 1970s and since then, it has been cultivated in small pockets of the country in different agroecology. It is a staple food in the country's east, where rice is imported through Somalia on the black market. Following the growing population of the country, rice demand increased, especially from city dwellers, forcing the government to spend large amounts of currency on importing rice [4]. This rise in demand combined with the increased import price, and availability of potential agroecology for rice production, challenged the country's policymakers to seriously think about the country's potential to grow the grain for its self-sufficiency. Consequently, successful arguments have pushed rice to be classified as a fourth "National Food Security Crop" after wheat, maize, and tef [4].

Along with this action the Ethiopian government set agricultural development policies and strategies and also has been heavily investing in infrastructure, rural finance, research, access to improved technology and information, market development, agricultural extension services, promotion of cooperatives, education, and resettlement programs. Following these interventions, the country achieved remarkable rice production growth in a short period. Rice-producing farmers also increased from 32, 000 in 2006 to 119, 000 in 2013, area allocated increased from 6,000 ha in 2006 to 58,000 ha in 2013 and production increased from 11,000 tons in 2006 to 184,000 tons [5, 6].

Although rice was introduced to Ethiopia very recently, rice production revealed increasing trend in area and productivity. In short time period it has proven to be a crop that can assure food and livelihood security [7]. Currently, rice in Ethiopia is considered as a strategic food security crop that has received due emphasis in the promotion of agricultural production, and as such it is considered as the "millennium crop" expected to support in ensuring food security in a country. The volume of rice production in Ethiopia is also moving with the national average productivity rise of 1.8 tons per hectare [8] to 2.8 tons per hectare [9] at an on-farm level as cited by [10]. However, it is argued that rice remains a minor crop regarding area coverage and production as compared to potential large areas and favorable agroclimatic conditions for expanding rice production.

Despite rice production being a profitable farm business offering up to 4038 ETB ha-1 [11], different farm-level production constraints limit its production and market supply. The reason for this gap was that the rice sector still faces challenges such as high competition with imported rice, poor infrastructure, insufficient mechanization, and post-harvest processing technologies, lack of skilled manpower and research facilities, and poor marketing infrastructure and channels [7]. Similarly, [12] also revealed that production constraints in rice crops are related to technical inputs; including improved seed availability, quality of improved varieties, fertilizer availability, and price, credit availability to buy fertilizer, and improved rice seeds. On the other hand, demographic and socio-economic factors including household labor availability, land holding, distance to the nearest village market, access to agricultural extension, access to the source of rice seeds, and access to new cultivars of rice affect technology adoption.

According to [13], constraints for sustainable production and productivity in rice were: poor access to improved rice varieties, especially for irrigated rice, and limited participation of seed growers in the production and marketing of rice seed; poor access and use of modern post-harvest techniques and equipment; grassy weeds and insect pests; limited access to credit; shortage of labor, specifically during periods of intensive weeding; poor knowledge of producers and other market actors about rice product quality; excessive numbers of intermediaries, and price seasonality; and inadequate storage facilities. To have increased rice yield the use of improved rice varieties is needed; however, income from rice production, farmers' membership in the organization, access to rice seed, cost of seed, yield, and level of training influenced the adoption of improved rice varieties [14].

As [15], puts ranges of major rice production challenges in Ethiopia into three categories. First, agronomic constraints include weeds, insect pests, diseases, poor land preparation, and limited utilization of chemical fertilizer. Secondly, pre-and post-harvest handlings problems like lack of thresher, rice mill, improved storage, and delayed harvesting and shattering. Thirdly, the authors added that the lack of effective extension packages was also challenging rice production in the country. Even though industrial fertilizer

application increases rice yield [16], the lack of effective fertilizer application affects the production and productivity of rice [12].

Different factors affect the market supply of rice. [17] reported that, in the Amhara region of Ethiopia, education level and quantity of rice produced affect the volume of rice sales positively but family size determines the volume of sales negatively and quantity produced jointly affected both the probability of market participation and volume of supply positively. The gender of the household head, access to improved seeds, years of formal education, and average rice yield influenced the probability that a farmer would participate in the market [14]. Southern Nations Nationalities and Peoples Regional State (SNNPRS) is the third largest producer of upland rice next to Gambella and Tigray in terms of estimated area coverage. In the region, rice is principally cultivated in Gimbo (Keffa), Guraferda, Menit Goldia, and Menit Shasha (Bench Maji), and Yeki (Sheka) districts [10]. From the major producing area in the region, Guraferda district is a major rice growing area in the Bench Maji zone particularly and SNNPR at large [18].

There were various previous studies conducted on rice in the district. According to a [18, 19] study, rice is one of the major cash crops in Guraferda District as all producer farmers supplied rice produce to the market in the production season. Their study mainly focused on marketing aspects giving little focus on production-related constraints. The [20] study assessed the economic efficiency of smallholder farmers in rice production in the district. However, their findings solely focused on measuring efficiency level and its determinants, showing little about production status and marketing-related problems. A study by [21] on production expansion and comparative advantage of upland rice production and its effect on the local farming systems showed better production status of rice in the district. However, rice marketing-related factors have not been assessed intensively. This study would add value by assessing production and marketing-related issues together. Therefore, this study was conducted with specific objectives to identify factors affecting smallholder farmers' market supply of rice and to assess constraints related to rice production and marketing in the district.

## 2. METHODOLOGY

### 2.1 Description of the Study Area

The study was undertaken in a major rice-growing area of the Guraferda district in the Bench Maji zone of SNNPR, southwest Ethiopia. Guraferda district is located at longitude 34088' - 36014'E and latitude 50033'-70.21'N [19]. It has a population of about 43,253 persons, of which 23,473 are males and 19,664 are females [9]. It is bordered on the south by the Bero district, on the west and north by the Gambela Region, on the northeast by Sheko, on the east by South Bench, and on the southeast by Menit Shasha. The estimated area of Guraferda district is about 2565.42 km2. There are 27 kebeles in the district. Based on the data obtained from the Agricultural and Rural Development Office of Guraferda, the population of the district was expected to exceed 45,028 [20]. In the district, agriculture forms the major livelihood as crops grown were rice, sorghum, maize, sesame, haricot bean, coffee, ground nut, spices like pepper, fruits such as orange, mango, papaya, and vegetables like banana, potato, cabbages, etc. Rice, sorghum, maize, and coffee were grown to a larger extent [20]. The altitude of the district ranges from 814 to 1995 meters above sea level. The temperature ranges between $10^{o}$c to $30^{o}$c. The mean annual rainfall is 1710 millimeters [19].

### 2.2 Sampling Techniques and Sample Size Determination

Two-stage sampling strategy was employed. Primarily, four sample rice producing *Kebeles* were selected randomly as they represent the whole district. In the second stage, by taking the list of rice producer household heads in each kebele, representative sample size was determined using the formula which was developed by Yemane cited in [21]: $n = \frac{N}{1+N(e)^2}$ where, n is sample size; N is target population, and e is level of precision. Based on this formula, by assuming level of precision 9%, and given number of total rice producer households in the district, sample size was estimated to be about 119.

$$n = \frac{3296}{1+3296(0.09)^2} = 119$$

## 2.3 Data Sources and Methods of Collection

In this study, data has been collected from both primary and secondary data sources. Primary data mainly collected regarding the demographic and socio-economic profile of rice producers. A structured questionnaire was used to generate the primary data from the selected sample of smallholder rice producers. The primary data were collected from the sample respondents and focus group discussions (FGDs) and key informants' interviews were also used to gather necessary information to supplement data collected from selected respondents. Secondary data considered to be important were collected from different sources including unpublished administrative documents of different organizations and published sources.

## 2.4 Method of Data Analysis

To realize the objectives of the study, two analytical tools were employed, namely, descriptive and econometric models.

### Descriptive Statistics

The sample respondents' demographic and socio-economic conditions were analyzed using descriptive statistics like mean, standard deviations, frequency, and percentage by using Statistical Package for Social Science (SPSS).

### Multiple Linear Regression Model

To analyze determinants of market supply of rice multiple linear regression model was used. The model was found to be appropriate because all the sample respondents supplied positive amount of rice to the market. The multiple linear regression model was specified as follows.

To analyze determinants of market supply of rice multiple linear regression model was used. The multiple linear regression model employed was specified as shown below.

$$Y_i = \beta_0 + \beta_1 SEX + \beta_2 AGE + \beta_3 HHS + \beta_4 FASIZE + \beta_5 RPRODCD + \beta_6 CREDT + \beta_7 INCOM + \beta_8 MARKET + \beta_9 EXTEN + \beta_{10} FERTLZR + \beta_{11} SEED + \beta_{12} EDUCTN + \beta 13 OXEN + \mu$$

Where, Yi is, market supply of paddy rice, β0 is intercept, β1-β13 denotes, coefficients to be estimated in relation to associated explanatory variables, SEX is sex of household heads, AGE is age of respondents, HHS is household size, FASIZE is total farm size, RPRODCD is rice quantity produced in a year, CREDT is credit used by a households, INCOM is annual income, MARKET is distance from nearest market, EXTEN is extension contact, FERTLZR is use of inorganic fertilizer, SEED is use of improved rice varieties, EDUCTN is education level, OXEN is, number of oxen owned and μ is Random error term.

Table 1: Summary of Definitions of Variables and Working Hypothesis

| Variables | Descriptions | Types | Expected sign |
|---|---|---|---|
| SEX | Sex (male=1 and female=o) | Dummy | + |
| AGE | Age of household in years | Continuous | + |
| EDUCTN | Education level (in years) | Continuous | + |
| HHSIZE | House hold size | Continuous | - |
| FARMICOME | Farm income (ETB in thousands) | Continuous | + |
| FARMSZ | Farm Size (in hectares) | Continuous | + |
| OXEN | Number of oxen owned | Continuous | + |
| RICEPRD | Quantity of rice produced | Continuous | + |
| MAKTDTC | Market distance (in Km) | Continuous | - |
| EXTN | Extension contacts (yes=1 and No=0) | Dummy | + |
| CREDIT | Credit use (yes=1 and No=0) | Dummy | + |
| FERTLZR | Use of inorganic fertilizers (yes=1 and No=0) | Dummy | + |
| IMPVDVATY | Use of improved varieties (yes=1 and No=0) | Dummy | + |

## 3. RESULTS AND DISCUSION

### 3.1 Descriptive Analysis

From the respondents 86.6% were married, 7.5 % were divorced and 6.7% were widowed. Most of the respondents (87.6%) were male-headed and 12.6 % were female-headed households. The average age of the respondents was 46.9 years with a minimum of 26 years and a maximum of 74 years. Farm activity was the basis for most farmers' livelihood as it was seen as a major source of household income and life support services. The average annual income of the respondents is 20,983 birrs (Ethiopian currency), which was mainly derived from agriculture. Farmers on average hold land size of 2.14 hectares. The mean yearly production of rice at the household level reaches one hectare with a mean production of nearly 2.8 tons per hectare. Farmers sold on average 70 % of their produced paddy rice to grain traders at the village level or district level; while value addition activities, like selling milled rice were not practiced by farmers.

Table2: Descriptive analysis of continuous variables

| Parameter | Minimum | Maximum | Mean | Std. Deviation |
|---|---|---|---|---|
| Age | 26.00 | 74.00 | 46.91 | 10.351 |
| Education level (in years) | 0.00 | 9.00 | 1.41 | 2.536 |
| Household size | 2.00 | 12.00 | 6.13 | 2.017 |
| Annual income (ETB in thousands) | 2.00 | 60.00 | 20.983 | 11.94 |
| Total farm size (hectares) | 0.25 | 6.00 | 2.2941 | 1.18 |
| Rice quantity produced (in tons) | 0.40 | 8.30 | 2.80 | 1.60 |
| Rice sold (in tons) | 0.00 | 7.50 | 1.91 | 1.48 |
| Oxen owned | 0.00 | 4.00 | 1.41 | 0.942 |
| Market distance (km) | 3.00 | 18.00 | 9.36 | 3.82 |

## 3.2 Determinants of Market Supply of Rice

The linear regression model results indicated in Table 3 below show that the supply of rice to the market was determined by the interaction of different demographic, economic, and institutional factors. Before conducting regression, the correlation of dependent variables and independent variables was checked and those variables satisfying the correlation conditions were included for regression analysis. The presence of multicollinearity among explanatory variables was checked and there was no serious problem. From the total 12 explanatory variables included in the econometric model, five variables were found to have a significant influence on the market supply of rice. These are household size, the quantity of rice produced, total farm size, total farm income, and credit use.

Farm size relation with sales volume was positive and influenced the marketable supply of rice positively at a 5 % level of significance. This shows that if the farm size of a household increases by a unit or simply by a hectare, the supply of rice to the market increases by 0.097 tons holding other things constant. This can be explained by the fact that farm households that have larger land sizes produce rice in larger amounts and have the probability to supply more. In addition, through diversification, they produce other crops like maize, sorghum, and others that can support household consumption needs, and since rice has a better market price than other crops, more rice is supplied to the market. The land is an important factor in production and the larger the size of productive land the producer owns, hence the higher the production levels are likely to be due enabling a household to produce a market surplus and be gifted to sell a substantial amount of produce [22].

Oxen owned was hypothesized to have a positive influence on the dependent variable and in the regression model it has shown a positive sign on the coefficient and the variable influenced the market supply of rice positively at a 1 % level of significance. This shows that if the number of oxen owned by a farmer increases by a unit, the supply of rice to the market increases by 0.15 tons holding other things constant. Since land preparation in the district is merely done by using oxen, having more of it enables producers to prepare land

on time and effectively to produce rice in larger plots and supply more to the market. The finding is similar with previous study by [18].

The quantity of rice produced affected sales volume in a positive relation and influenced the market supply of rice at a 1% level of significance. This shows that if the quantity produced increases by a unit, the supply of rice to the market increases by 0.063 tons holding other things constant. Rice in the district was produced in a better market-oriented way than other crops. It can be explained by a farmer who produces more would probably supply more to the market. In line with this finding, [23] also showed higher yield increases the farmer's likelihood to participate in the market because the surplus above their household consumption needs makes more supply to the market. The quantity of rice produced is positively related to the intensity of market participation as the quantity produced is critical for semi-commercial farmers who first have to produce for home consumption and only sell surplus [22].

Credit use influenced the market supply of rice positively and significantly at a 5 % level of significance. This shows that if households participated in credit, the supply of rice to the market increased by 0.16 tons holding other things constant. This might be due to the reason that rice producer households with labor and financial shortages need to use hired labor for land preparation, weeding, and harvesting, as a result, they utilize credit for paying labor and fertilizer costs for rice production in larger amounts. In line with this, other researchers also showed that access to credit enables producers to increase the amount of inputs and other inputs (fertilizer, seed, plows) which in turn boosts output produced and surplus for the market [22].

Total farm income influenced the market supply of rice positively at a 1 % level of significance. This shows that if households' annual income (in thousands), increases by a unit, the supply of rice to the market increases by 0.02 tons holding other things constant. It might be due to a farmer with a better annual income having a better opportunity to produce rice in larger size by employing all necessary production inputs and supplying more rice to the market than those with lower income.

Table 3: OLS regression results for sales volume of rice

| Variables | Coefficient | Std. Error. | t-ratio |
|---|---|---|---|
| Sex of household head | -0.015 | 0.102 | -0.15 |
| Age of household head | -0.004 | 0.004 | -0.91 |
| Education level | 0.001 | 0.013 | 0.72 |
| Household size | 0.031 | 0.021 | 1.45 |
| Farm size | 0.097 | 0.042 | 2.27** |
| Rice quantity produced | 0.063 | 0.004 | 15.33*** |
| Fertilizer application | 0.063 | 0.065 | 0.95 |
| Improved variety use | 0.067 | 0.094 | 0.70 |
| Distance to the market | -0.001 | 0.0088 | -0.21 |
| Extension contacts | -0.053 | 0.081 | -0.66 |
| Credit use | 0.168 | 0.079 | 2.13** |
| Farm income | 0.021 | 0.004 | 4.73*** |
| Oxen number owned | 0.150 | 0.057 | 2.66*** |
| Constant | -0.764 | 0.21 | -3.62 |

Number of observations = 119 R-squared = 0.954
Root MSE = 3.385 Adj R-squared = 0.9484
F (13, 106) = 167.67 Prob.> F = 0.0000

**3.3 Constraints Related to Rice Production in the District**

**Inorganic fertilizer application**

Most of the respondent farmers mentioned that the specific time of fertilizer application, type and amount as well as way of application was not clearly known and, in the event, that unless these conditions were fixed, they believe that fertilizer application contributed no significant yield difference. Due to this, some farmers were hesitant to apply inorganic fertilizer on rice as they believed that there was no positive significant change in the yield. Even though some farmers accept extension agents' advice on the provision

and application of inorganic fertilizer to rice fields, some of them use it inappropriately seemingly showing unnecessary resource wastage. According to [24], the efficiency of nitrogen applied to rice crops depends on the type of fertilizer, timing of application, seasonal trends, and others related factors. However, it needs critical care during decision making in relation to rate, application date and crop growth stage. However, in Ethiopia, there is a gap in conducting in-depth research studies to improve the grain yield except few studies done on different N rates on a few rice cultivars. According to [25] rice yield in the district is low due to lack of area-specific recommendations on fertilizer rate.

**Weak crop management practice**

Rice needs higher management practice especially, at the time of weeding than other major crops grown in the area. For farmers who produce it intensively, weeding becomes a difficult task as weeding is done by hand. Reports showed that weed is a major problem in rice production in Ethiopia as it is a labor-intensive crop, in which about 67% of the cost expenditure goes for weeding purposes, extending up to 2939.41 birr per hectare [17]. Despite most of the farmers in the area using herbicide for weed control, its ineffectiveness on grassy weed and Mimosa invisa weed made hand weeding the only option with intensive labor force employment. [26] indicated that the rice yield losses due to weed interference in the district can reach 68%. In addition, rainfall shortage during a critical rice growing period was affecting yield highly and this shows that the issue needs further soil and water management practices and variety selection for moisture stress tolerance.

**Weak institutional support**

Problems related to security problems sometimes cause conflict, extension service provision, weak research-extension-farmer linkage, administrative issues related to land ownership certification, and weak monitoring of traders and markets by concerned government organizations were problems farmers pointed out. Strong institutional support along the value chain of rice contributes to effective value chain development for the commodity. Farmers' smooth participation along different value chain actors like

research organizations, cooperatives, extension, and other development associations including NGOs is necessary to improve farmers' rice production experience [27]. However weak institutional support in the district was mentioned as one of the major challenges in the district.

**Improved seed supply shortage**

Most of the farmers were using well-known local rice varieties and little improved varieties. However, farmers need high-yielding improved varieties critically. According to the farmers' response, the existing newly supplied rice varieties, especially, NERICA-4 have a threshing problem after harvesting, unlike the local one which is easily threshed immediately after harvest. In addition to this, NERICA 4 has a short height characteristic and it is incompatible with the local farming system. As a result, compared to local varieties the improved varieties were not adopted by most of the producers yet. The need for other promising new varieties is continued as it holds a high-value question for researchers to provide varieties to increase productivity sustainably in the district. [14] revealed the contribution of access to improved rice varieties via that adoption helps to participate in rice markets and that in turn had a higher significant impact on welfare indicators; such as consumption expenditure, rice income, average yield, and access to credit.

**Lack of effective extension service support**

In the area, the extension services are limited only to fertilizer supply, rather there was a weak focus on agronomic management practices, crop protection, and marketing services. Due to this reason, some farmers face a knowledge gap in agronomic crop management practices. Crop and animal production with frequent and effective monitoring and support were some of the major provisions that farmers need from extension service providers. Extension service based on farmers' participatory research and training programs contributes to increased dissemination of information and demonstration and popularize new rice technologies; showing the need to consider farmers' preferences for new varieties and others through research and demonstrations at farmers' fields to enhance and promote the adoption [28].

**Disease and pest problem**

According to the sample farmers' response, one of the most important related rice production constraints was disease. Farmers identified it locally known as "MICHI" which causes a high amount of yield loss. The symptoms of the disease that respondents mentioned were early related to that of rice brown spot. However, the prior research finding of [29] in the Guraferda district also revealed, that higher incidence and severity of rice blast has been recorded in the district. Some research findings showed that the low productivity of rice in Ethiopia is attributed to several factors, important of which are seed-borne diseases, causing 50-80 percent yield losses. Among these, blast disease was reported in almost 85 rice-growing countries of the world. According to [30] fungi, viruses, bacteria, insects, and nematodes are causing problems in rice crop and have potential to affect production and productivity if appropriate solution not provided timely.

**Price setting & scaling problem**

Most farm households use rice as a cash crop and they cover their cash needs for fertilizer costs, oxen buying, schooling costs, and others by selling at the time they need. Respondents said that some traders communicate with each other and make the rice price lower at the time when they bring the rice to the market and that affects the bargaining power of the farmers. This condition makes farmers sell their produce at a lower price than bringing the product back to the home if cash needs are urgent. The market related problems faced by farmers in marketing their output is due to the lack of clear information about the price level of product in the market as the price is easily manipulated by traders [31].

**Shortage of threshing and milling machine**

Due to the unavailability and high cost of milling machines, all the farmers are selling their rice in the form of paddy and this incapacitates farmers to benefit from better prices by adding value to their product. The difficulty of threshing for the NERICA-4 variety as a problem was mentioned by the producers which was one of the main reasons for the lower adoption of the variety.

**Lack of market**

The absence of better competition among traders in the district narrowed the alternative possibility of getting better prices at better markets for rice output. However, some produces mentioned the situation is better compared to what was happening several years back.

Table4: Constraints related with rice production

| What are constraints in rice cultivation? | Yes (N=119) | Percent | St. Deviation |
|---|---|---|---|
| Lack of market and market information | 22 | 18.6 | 0.390 |
| Fertilizer application &price | 90 | 75.6 | 0.431 |
| Improved seed supply shortage | 60 | 50.4 | 0.502 |
| Land shortage | 28 | 23.5 | 0.426 |
| Disease and insect pest occurrence | 48 | 40.3 | 0.493 |
| Transport shortage | 15 | 14 | 0.409 |
| Institutional support problem | 87 | 73.1 | 0.445 |
| Extension service provision | 64 | 53.8 | 0.501 |
| Price setting & scaling problem | 70 | 58.8 | 0.494 |
| Crop management | 69 | 58 | 0.482 |

## 4. Concluding remarks

In the district, most smallholder farmers are producing rice in a better market-oriented way than other crops grown in the area and it created better employment opportunities for most farm households. Rice was being produced as a cash crop by most farmers as they supplied more of their product (more than 68%) to the market to make money for farming and household consumption expenditures. Determinants of the market supply of rice were total farm size, total household size, credit use, total farm income, and quantity of rice produced.

Major problems related to rice production in the area were a lack of improved rice production technologies, the requirement of heavy crop management especially weeding, fertilizer supply and high prices, problems related to security problems sometimes causing conflict, extension service provision, weak research-extension-farmer linkage, administrative issues related with land ownership certification, weak monitoring of traders and market by concerned government organization. The rice crop is a significant contributor and the same will be in the future as the crop has considerable potential to improve the livelihood of farm households and communities. Thus, attention should be given to rice production and marketing-related constraints.

Despite agricultural production increases through either an increase in farm size or the application of more modern agricultural inputs, high population pressure makes the application of modern technologies the only solution. Rice is a major crop that can be considered as a base for the livelihoods of households in a district, improved rice technologies are necessarily needed to benefit smallholder farmers. Thus, attention should be given to rice production-associated constraints. Introduction, evaluation, and generation of improved high-yielding rice varieties and other demand-driven rice technologies for producers should be provided by concerned government and nongovernmental organizations via strong research extension and farmer linkage. Soil fertility with soil and water management practices should get attention as it is necessary to counterbalance adverse weather conditions and that should be supported with a shift to using short-growing and moisture stress-tolerant rice varieties generation and expansion.

Since rice production requires heavy management practices and credit use has shown a positive relation with marketed rice supply, rural finance outreach and access to every smallholder farmer need to get attention to increase rice production and market supply. Small family-sized households with financial shortages face labor shortages for rice production, especially at the weeding time, and consequently, their marketed rice supply becomes lower and they need to be supported by improved crop management technologies especially, weed control systems.

Having more oxen showed more rice production and increased supply to the market. But, disease and other problems like rustling challenge livestock keeping. Therefore, disease prevention and management mechanisms should be strengthened. In addition, introducing and encouraging capable farmers to use other improved land preparation technologies like machinery(tractor) is needed and better if got attention from concerned bodies.

Most extension agents joining the rice producer community after graduation from college have weak practical experience in rice production. Therefore, building the capacity of extension agents through effective training should get attention to increase production and productivity including improvement in the quality of rice products through better post-harvest handling and processing.

## ACKNOWLEDMENTS

The author would like to acknowledge Southern Agricultural Research Institute for the financial and overall facilities provided during the execution of this study.

## Conflict of Interest

The author does not have any conflict of interest.

## REFERENCES

1. Food and Agriculture Organization of the United Nations (FAO). Rice Market Monitor. Food and Agriculture Organization of United Nations. 2017; XX (2).
2. Hegde S, Hegde V. Assessment of global rice production and export opportunity for economic development in Ethiopia. Int. J. Sci. Res. 2013; 2:257-260.
3. Wang C, Liu X, Peng S, Xu Q, Yuan X, Feng Y, Yu H, Wang Y, Wei X. Development of novel microsatellite markers for the BBCC Oryza genome (Poaceae) using high-throughput sequencing technology. PloS One. 2014;9(3):91826.
4. Negussie T, Alemu D. An Overview of the National Rice Research and Development Strategy and its Implementation. Challenges and Opportunities of Rice in Ethiopian Agricultural Development. 2011;1-16.
5. Kebede E. Trends and challenges on Rice investment in Ethiopia. Challenges and Opportunities of Rice in Ethiopian Agricultural Development. Empowering Farmers Innovation. EIAR/FRG II, Addis Ababa, Ethiopia. 2011;2.

6. Central Statistical Agency (CSA). Agricultural sample survey. Report on area and production of crops (Private peasant holdings, Meher season) Central Statistical Agency of Ethiopia, Addis Ababa, Ethiopia; 2016.

7. Misganaw Anteneh Tegegne. Assessment on Constraints and Opportunities of Rice Production Trends in Fogera - Ethiopia. The Case of Lowland Rice Production System", International Journal of Research Studies in Agricultural Sciences (IJRSAS), vol. 6, no. 4, pp.1-7, 2020. Available: DOI: http://dx.doi.org/10.20431/2454-6224.0604001

8. Central Statistical Agency (CSA). Agricultural sample survey. Report on area and production of major crops. Central Statistical Agency of Ethiopia, Addis Ababa, Ethiopia; 2005.

9. Central Statistical Agency (CSA). Population Projection of Ethiopia for all Regions at the Wereda Level from 2014 – 2017. Addis Ababa, Ethiopia; 2013.

10. Abayneh KK, Addis AT, Altaye TG. Estimation of Genetic Variability, Heritability, and Genetic Advance in Upland Rice (Oryza Sativa L.) Genotypes at Guraferda, South West Ethiopia. Journal of Food Science and Quality Management. 2018; 72:2225-0557.

11. Yalfal T, Daniel T, Bayuh B. Production expansion competitiveness and comparative advantage of upland rice production: The case of Fogera and Libokemekem Plain in Ethiopia. The International Journal of Applied Economics and Finance; 2014.

12. Afework Hagos. Choices of Varieties and Demand for Improved Rice Seed in Fogera District of Ethiopia. Research Thesis Submitted to Haramaya University; 2014.

13. Tamirat Belayneh, Jember Tekle. Review on adoption, trend, potential, and constraints of rice production to livelihood in Ethiopia International Journal of Research - Granthaalayah, 2017;5(6):644-658. Available: https://doi.org/10.5281/zenodo.824116

14. Awotide BA, Karimov AA, Diagne A. Agricultural technology adoption, commercialization and smallholder rice farmers' welfare in rural Nigeria. Agricultural and Food Economics, 2016;4(1):3.

15. Ayele A. Rice Extension approaches and extension packages in Ethiopia. Empowering Farmers' Innovation. Addis Ababa. 2012;4EIAR/ FRG II.

16. Rugumamu CP. Empowering smallholder rice farmers in Tanzania to increase productivity for promoting food security in Eastern and Southern Africa. Agriculture & Food Security. 2014;3(1):7

17. Takele A. Analysis of Rice Profitability and Marketing Chain: The Case of Fogera Woreda, South Gondar Zone, Amhara National Regional State, Ethiopia. M.Sc. Thesis submitted to Haramaya University; 2010.

18. Agegnehu Workye, Degye Goshu, Bosena Tegegne. Analysis of Factors Influencing Market Supply of Rice by Smallholder Farmers in Guraferda District, Southwest Ethiopia. Agriculture, Forestry and Fisheries. Vol. 8, No. 5, 2019, pp. 95-99. https://doi:10.11648/j.aff.20190805.11

19. Workye A, Goshu D, Tegegne B. Analysis of Factors Influencing Market Supply of Rice by Smallholder Farmers in Guraferda District, Southwest Ethiopia, Agriculture, Forestry and Fisheries. 2019;8(5)95-99: https://doi.org/10.11648/j.aff.20190805.11

20. Melese T, Alemu M, Mitikuand A, Kedir N. Economic Efficiency of Smallholder Farmers in Rice Production: The Case of Guraferda Woreda, Southern Nations Nationalities People's Region, Ethiopia. International Journal of Agriculture Innovations and Research. 2019;(8) 2:2319-1473. (Online)

21. Mulatu E, Alemayehu A. Production Expansion and Comparative Advantage of Upland Rice Production and its Effect on the Local Farming Systems: The Case of Guraferda District, Southwest Ethiopia. Adv Crop Sci Tech. 2018; 6:395. DOI: https://doi.org/10.4172/2329-8863.1000395

22. Moono L. An Analysis of Factors Influencing Market Participation Among Smallholder Rice Farmers in Western Province, Zambia. A Thesis Submitted Department of Agriculture Economics Faculty of Agriculture University of Nairobi; 2015.

23. Esther LA and Gaudiose M. Market participation by smallholder rice farmers in Tanzania: a double hurdle analysis. Studies in Agricultural Economics 2017; 118:112-115. Available: http://dx.doi.org/10.7896/j.1528

24. Getachew M, Nebiyu A. Nitrogen Use Efficiency of Upland Rice in the Humid Tropics of Southwest Ethiopia in Response to Split Nitrogen Application. Journal of Agronomy. 2018;17(1)68-76.

25. Abriham, A. and Nazib, N. Response of rice to nitrogen rate and inter-row spacing at guraferda and gojeb south western Ethiopia. International Journal of Agricultural Extension, 10(1), pp.41-46. https://doi.org/10.33687/ijae.010.01.3682

26. Mekonnen G, Woldesenbet M, Yegezu E. Determination of Critical Period of Weed-Crop Competition in Rice (Oryza sativa L.) in Bench Maji and Kaffa Zone, South Western Ethiopia. Journal of Plant Sciences. 2017;5(3)90-98: https://doi.org/10.11648/j.jps.20170503.11

27. Rugema, SH, Sseguya H, Kibwika P. Determinants of smallholder farmers' participation in rice value chains in Uganda. Journal of Agricultural Extension. 2018;22(2).

28. Ghimire R, Wen-chi HUANG, Shrestha RB. Factors affecting the adoption of improved rice varieties among rural farm households in Central Nepal. Rice Science, 2015;22(1):35-43.

29. Jember MB. Genetic variability and associations among yield and yield-related traits in selected upland rice (Oryza sativa L. and Oryza glaberrima Steud) Genotypes. M.Sc. Thesis Submitted to Haramaya University; 2015.

30. Melkamu Andargie, Merkuz Abera, Adane Tesfaye & Esuyawkal Demis. Occurrence, distribution, and management experiences of rice (Oryza sativa L.) major diseases and pests in Ethiopia: a review, Cogent Food & Agriculture, 10:1, DOI: https://doi.org/10.1080/23311932.2023.2300558

31. Nurhalisa, N., Helviani, H., Masitah, M., Nursalam, N. and Bustang, B. Rice Marketing Channels. Agribusiness Journal, 6(1), pp.1-4. DOI: https://doi.org/10.31327/aj.v6i1.1837

Click here to access/download
**Other**
PLOSOne_Human_Subjects_Research_Checklist.docx
