# Supplementary material for: Determinants of rice production and market supply: A study of Bench Sheko zone in Ethiopia
Source: PLoS One. 2024 Sep 6;19(9):e0302115. doi: 10.1371/journal.pone.0302115 (PMC11379224; doi:10.1371/journal.pone.0302115)
Supplement: Attachment — Submitted filename: Response to Reviewers.pdf [file pone.0302115.s003.pdf]

Dear,

Meraj Alam Ansari
Academic Editor, PLOS ONE

Thank you for giving me the opportunity to submit a revised manuscript titled: Factors Affecting Rice Production and Market Supply: the case of Guraferda district, Bench Maji zone in Southern Ethiopia to PLOS ONE Journal.

I appreciate your time and effort that journal editor and the reviewers have dedicated to providing your comment to modify the manuscript. I am really grateful to the editor and reviewers for their insightful comments on improving my paper and make sound. I have been able to incorporate all necessary comments and suggestions provided by the editor and reviewers. I have made the changes within the manuscript with the track change and submitted with also the revised manuscript for publication. Herewith I have provided response to all the relevant reviewers' comments.

Comments from Editor
Comment1: Change the title its looking local study, it should be region specific title Determinants of Rice Production and Market Supply: A Study of Bench Maji Zone, Ethiopia"
Response: Accepted and modified the title accordingly

Comment 2: *Abstract should be supported with quantified data of your results.*
Response: Accepted and modified with thanks

Comment 3: *Define all the determinants taken in this study and support with quantified data as well as references.*
Response: All available reviews were done and all determinants defined

Comment 4: *Introduction is too long and vague statements must be discarded from introduction. Focused introduction should be rewritten.*

Response: This comment has been taken as a very critical and all necessary adjustments were made and introduction part made more condensed and shortened.

Comment 5: *Study area map should be included*
Response: Accepted and map is included

Comment 6: *Conclusion looks like a thesis finding. It should be very sound and brief. Policy implication should be added separately.*
Response: Accepted and made brief

Comment 7: *Check your references properly, excess reference should be deleted and missing references in text should be added in the list.*

Response: Reference part has been double checked and necessary adjustments done
Comment 8: Punctuation and grammatical error should be rectified throughout the manuscript.

Response: Checked thoroughly the paper and modified

Comments from the reviewer 1: *The manuscript must describe a technically sound piece of scientific research with data that supports the conclusions. Experiments must have been conducted rigorously, with appropriate controls, replication, and sample sizes. The conclusions must be drawn appropriately based on the data presented.*

Response: As the study was based on household survey, there is no experiment conducted and conclusion drawn based on the data presented.

Comments from the reviewer 4: *Any typographical or grammatical errors should be corrected at revision, so please note any specific errors here.*

Response: Accepted and all necessary modifications made

Comments from reviewer 5:

Comment 1. *The title should clearly reflect the specific focus of the study. Consider revising to something more precise, such as "Factors Influencing Smallholder Farmers' Market Supply of Rice in Guraferda District, Ethiopia."*
Response: As the study was mainly focused on market supply and production, the author wanted to mention the title as the editor commented.

Comment 2. *Language and Grammar: Revise for grammatical accuracy and readability.*

Response: Accepted and all necessary modifications made

Comment 3. *The results can be improved by adding figures.*

Response: depending on the type of study, could not find appropriate figure to add. Only map of the study area added on methodology part.

Comment 4. *Comparing and contrasting with similar studies would add depth to the discussion.*

Response: Accepted and all necessary modifications made

Sincerely,

Ejigu Mulatu
