# Supplementary material for: Determinants of rice production and market supply: A study of Bench Sheko zone in Ethiopia
Source: PLoS One. 2024 Sep 6;19(9):e0302115. doi: 10.1371/journal.pone.0302115 (PMC11379224; doi:10.1371/journal.pone.0302115)
Supplement: Attachment — Submitted filename: Response to Reviewers.docx [file pone.0302115.s004.docx]

**Dear,**

**Meraj Alam Ansari
Academic Editor, PLOS ONE**

Thank you for giving me the opportunity to submit a revised manuscript titled: determinants of rice production and market Supply: the case of Bench Maji zone in Ethiopia to PLOS ONE Journal.

I appreciate your time and effort that journal editor and the reviewers have dedicated to providing your comment to modify the manuscript. I am really grateful to the editor and reviewers for their insightful comments on improving my paper and make sound. I have been able to incorporate all necessary comments and suggestions provided by the editor and reviewers. I have made the changes within the manuscript with the track change and submitted with also the revised manuscript for publication. Herewith I have provided response to all the relevant reviewers’ comments.

**Comment on reference**
**Comment1**: Follow journal reference style and make complete and correct. Comment on retracted articles

**Response**: Accepted and modified all the references used. All relevant changes were made. I have not used retracted articles.

**Additional Editor Comments:**

1.Introduction section may focused with inclusion of Keywords, gap, novelty of study, robust hypothesis etc.

**Response**: The comment is accepted and all necessary improvements were made.

2. References must be arranged in the format of PLOS One. In refernce No. 14,22, 27, 28 year of publication is missing.

**Response:** The comment is accepted and all necessary improvements were made. Major revision in reference section made and hopefully fits the journal requirement. Thank you for this very important and critical comment.

Sincerely,

Ejigu Mulatu
